# Determinants of financial inclusion gaps in Pakistan and implications for achieving SDGs

**DOI:** 10.1038/s41598-024-63445-6

**Published:** 2024-06-13

**Authors:** Amar Razzaq, Shengze Qin, Yewang Zhou, Irfan Mahmood, Mohamad Alnafissa

**Affiliations:** 1 Business School, Huanggang Normal University, No. 146 Xinggang 2nd Road, Huanggang, 438000 China; 2 School of Tourism Management, Wuhan Business University, 300 Dongfeng Blvd, Cai Dian District, Wuhan, 430118 China; 3 College of Economics & Management, Huazhong Agricultural University, No. 1 Shizishan Street, Hongshan District, Wuhan, 430070 China; 4 Pakistan Agricultural Research Council, 1 Ataturk Ave, G-5/1, Islamabad, 44050 Pakistan; 5 Department of Agricultural Economics, College of Food and Agricultural Sciences, King Saud University, P. O. Box 2460, 11451 Riyadh, Saudi Arabia

**Keywords:** Women’s financial inclusion, Gender equality, Digital financial services, Pakistan, Socio-economic development, Financial literacy, SDG#5, Environmental sciences, Environmental social sciences

## Abstract

This study investigates the determinants of gender disparities in financial inclusion in Pakistan using Global Findex 2021 survey data. We aim to quantify gender gaps in financial access and use, and to analyze the socio-economic factors influencing these disparities. Grounded in Sen’s capability approach and behavioral economics, we employ logistic regression to examine how gender influences the ownership and usage of financial products. Our results reveal significant gender gaps: only 13% of Pakistani women have financial accounts compared to 34% of men, with similar disparities in digital finance. Socio-economic variables like education, income, and employment are found to influence financial inclusion differently for men and women. While generally supportive of financial inclusion, these factors have a weaker effect for women, suggesting deeper societal barriers. This study adds to the global financial inclusion discourse by providing a comprehensive analysis of gender disparities in Pakistan. Our findings highlight the need for gender-sensitive policies that address these disparities to achieve Sustainable Development Goals related to gender equality and economic empowerment.

## Introduction

As the world strives towards achieving sustainable development and eradicating poverty, the importance of financial inclusion has gained significant recognition. Providing access to formal financial services is seen as a critical step in empowering individuals and communities, particularly in developing countries where large segments of the population remain excluded from the formal financial system. Financial inclusion, defined as access to and use of formal financial services, is recognized as a key driver of economic development and poverty reduction, particularly in developing countries like Pakistan^1–4^. It extends beyond mere banking services, encompassing digital payments, financial literacy, and access to diverse forms of insurance and credit facilities, particularly for the disadvantaged and low-income groups^5,6^. These services are essential for building economic security and fostering human development by integrating marginalized groups into the formal economy. This inclusive approach ensures economic security and promotes human and economic development by integrating underserved populations into the mainstream economy^7^. Moreover, financial inclusion is fundamental to achieving the Sustainable Development Goals (SDGs), specifically SDG 1 (No Poverty), SDG 5 (Gender Equality), and SDG 10 (Reduced Inequalities), by promoting sustainable livelihoods, enhancing economic resilience, and empowering individuals to invest in education and healthcare^8–11^. However, despite the recognized importance of financial inclusion and global efforts to promote it^9,10^, significant disparities persist, especially along gender lines. Women, in particular, continue to face disproportionate barriers in accessing and using financial services, hindering their economic empowerment and perpetuating gender inequalities.

In Pakistan, the gender gap in financial inclusion remains substantial, highlighting the need for targeted interventions and policies. This disparity not only hinders women’s economic empowerment and perpetuates gender inequalities but also has broader implications for the country’s overall economic development and achievement of the SDGs, particularly SDG 5 on gender equality. Women are significantly less likely than men to own or use formal financial products and services^12,13^. For instance, according to the Global Findex 2021 data, only 13% of women in Pakistan have a formal bank account, compared to 34% of men^14^. This disparity reflects and reinforces broader social and economic inequalities within the country^15,16^. Understanding the determinants of financial inclusion is essential for developing effective policies to address these disparities. Existing research has identified several factors influencing financial inclusion in Pakistan, including income levels, educational attainment, employment status, and urban/rural residency^17,18^. However, there is a lack of studies that specifically examine how these factors affect financial inclusion differently for men and women in Pakistan^19,20^. This gap in knowledge limits our understanding of the complex dynamics that shape women’s financial inclusion in Pakistan and hinders the development of effective, gender-sensitive interventions to address the unique challenges faced by women.

To effectively address this research gap and develop targeted interventions, it is important to understand the specific challenges faced by women in Pakistan that hinder their access to and use of financial services. These challenges include discriminatory social norms, limited access to education and employment opportunities, and legal constraints related to inheritance and property ownership^21–24^. For example, a recent study found that only 24% of women in Pakistan are employed, compared to 82% of men, and women’s labor force participation rate has remained stagnant over the past two decades^25^. The State Bank of Pakistan (SBP) has recognized the significance of these challenges and has taken steps to promote gender equality in financial inclusion through initiatives such as the National Financial Inclusion Strategy (NFIS) and the Banking on Equality (BOE) policy^26,27^. The BOE policy is based on five key pillars: (1) Gender Diversity in Financial Institutions and their Access Points, (2) Women-Centric Products and Outreach Targets, (3) Women’s Champions at All Customer Touch Points, (4) Robust Collection of Gender-Disaggregated Data and Target Setting, and (5) Policy Forum on Gender and Finance. This policy aims to reduce the gender gap in financial inclusion by introducing targeted measures to improve women’s access to and usage of formal financial services. While the government of Pakistan has implemented programs and policies aimed at promoting financial inclusion, these initiatives often fall short of addressing the specific needs and challenges faced by women^16,28^. This underscores the need for comprehensive research that examines how these factors interact and contribute to the gender gap in financial inclusion in Pakistan.

Addressing this research gap is critical not only for promoting gender equality and women’s empowerment but also for achieving broader development goals. The theoretical frameworks of Sen’s capability approach^29^ and behavioral economics^30^ provide valuable lenses for understanding the multi-dimensional nature of financial inclusion and its impact on individual well-being and societal progress. By examining how gender interacts with various socio-economic factors to influence financial inclusion outcomes, this study aims to contribute to the existing knowledge base and inform the design and implementation of more effective policies and interventions.

While acknowledging the valuable data collected by the SBP on gender and financial inclusion through its Access to Finance Survey (A2FS) of 2008 and 2015, the present study aims to provide an in-depth examination of the current state of gender disparities in financial inclusion in Pakistan. It draws on the latest data from the Global Findex 2021, which offers benefits in international comparability and comprehensive coverage of financial access and usage indicators^14^. The Global Findex is a comprehensive and internationally recognized dataset that provides detailed information on financial inclusion across 144 countries, making it an invaluable resource for analyzing gender disparities in financial access and usage. By analyzing gender-specific trends and determinants of both access to and usage of financial services, this research contributes to a more complete understanding of financial inclusion in Pakistan and informs the development of effective policies that promote women’s financial empowerment.

Grounded in Sen’s capability approach^29^ and principles of behavioral economics^30^, the contributions of this study are threefold. First, it provides a comprehensive and up-to-date analysis of the current situation of financial inclusion in Pakistan, with a specific focus on gender disparities. Second, it utilizes robust econometric methods to examine the determinants of financial product ownership and usage, differentiating between the experiences of men and women. This gender-disaggregated approach allows for a deeper understanding of the factors that contribute to the financial inclusion gap. Third, by identifying the specific challenges and opportunities associated with gender disparities in financial inclusion, this study provides valuable information for policymakers and stakeholders to develop targeted strategies and interventions to enhance financial inclusion and address gender inequalities in Pakistan. The findings of this study have the potential to inform the design and implementation of policies and interventions that promote women’s financial empowerment, thereby contributing to the achievement of the SDGs and fostering inclusive economic growth in Pakistan.

## Literature review

### Global trends in financial inclusion

The global financial landscape has been significantly shaped by the advancement in financial inclusion, especially facilitated by digitalization. This trend highlights the strong link between financial inclusion and key economic indicators such as Gross Domestic Product (GDP) per capita and employment rates, emphasizing the importance of banking competitiveness and the effectiveness of government policies^31^. Concurrently, the rise of digital finance has marked a new era characterized by low interest rates and economic growth, thereby widening opportunities for asset accumulation and financial accessibility^32^. Moreover, the emergence of open banking and the increasing use of artificial intelligence and machine learning in financial services are further revolutionizing the financial inclusion landscape, creating new avenues for reaching underserved populations and tailoring financial products to their specific needs^33,34^ Nevertheless, institutional factors such as the management of corruption and the efficacy of governance play an important role in advancing financial inclusion^35^.

Regionally, the impact of these developments has been diverse. For instance, in sub-Saharan Africa, significant increases in financial inclusion have been observed, with significant differences among countries^36^. In addition, the impact of Islamic banking on improving financial inclusion in nations such as Bangladesh and Malaysia indicates a progression toward more inclusive banking methods across diverse cultural settings^37^.

### Significance of financial inclusion in economic growth

The contribution of financial inclusion to economic growth and the alleviation of poverty is increasingly acknowledged. Its importance in minimizing social exclusion and bolstering economic growth aligns with the broader objective of inclusive growth^4^. Similarly, financial inclusion is argued to expand the resource base of the financial system, aiding in economic development and poverty alleviation^38^. Moreover, financial inclusion is essential for catalyzing economic expansion and reducing disparities in income levels. Its impact is notably significant in emerging economies, such as India, where it substantially aids in socio-economic progress^39,40^.

### Financial inclusion in Pakistan

In Pakistan, the trajectory of financial inclusion has been shaped by a range of socio-economic factors. Recent research has indicates a paradoxical increase in carbon dioxide emissions associated with financial inclusion, implying a complex relationship between financial growth and environmental goals^41^. This aspect has brought to the forefront the need for policy re-evaluation to integrate more environmentally friendly approaches. Further research suggests a direct impact of financial inclusion on the disposable incomes of Pakistan’s low-income urban households, highlighting its role in enhancing living standards and contributing to poverty reduction^17^.

The evolution of financial inclusion in Pakistan also reflects a significant shift. Initially centered on poverty alleviation, the country’s financial inclusion policy is increasingly aligning with broader monetary control objectives. This change represents a shift in national macroeconomic priorities^42^. Complementing this perspective, financial inclusion is viewed as a critical factor in poverty reduction, particularly in Pakistan’s Karakoram valleys. This emphasizes the regional differences in impact and the need for localized financial inclusion strategies^43^. The State Bank of Pakistan has taken significant steps to promote financial inclusion through its National Financial Inclusion Strategy (NFIS). The NFIS, launched in 2015, sets out targets and priorities for expanding access to and usage of formal financial services, with a particular focus on underserved segments, including women^27^. Under the NFIS, the SBP has set a target of ensuring that at least 20 million women have active digital transaction accounts by 2023. The NFIS and the “Banking on Equality” policy demonstrate the SBP’s commitment to addressing the gender gap in financial inclusion and promoting inclusive economic growth^26^.

### Socio-economic factors influencing financial inclusion in Pakistan

The factors contributing to financial inclusion in Pakistan are multifaceted and complex. Research indicates that rural communities and financial access techniques significantly influence financial inclusion in the country^20^. Furthermore, factors such as gender, income, education, and age have been identified as critical in shaping the landscape of financial inclusion in Pakistan^18^.

The role of socio-demographic and political factors in financial inclusion is particularly significant in contexts where economic development is limited. This perspective is asserted through studies that emphasize the significance of access, availability, usage, and cost as key drivers of financial inclusion. These factors are instrumental in enhancing the disposable income among low-income urban families in Pakistan^17,44^. In addition, research emphasizes the strong link between financial inclusion, financial development, and poverty reduction in Pakistan, demonstrating its importance for socio-economic progress^45^.

### Gender disparities in financial inclusion in Pakistan

Gender dynamics in financial inclusion in Pakistan reveal deep-rooted social and economic disparities. Educational disparities, particularly affecting women, have been shown to significantly impede their economic participation and growth. Gender educational disparities are a major obstacle to women’s educational attainment, thereby limiting their economic participation and affecting the country’s broader economic landscape^46^. Furthermore, a comprehensive analysis of gender inequality in Pakistan demonstrates that these disparities extend across various sectors, including political participation and economic activity, leading to pronounced social and economic inequalities^13^. Recent studies have also emphasized the role of intra-household bargaining power and decision-making dynamics in influencing women’s financial inclusion, suggesting that empowering women within households is critical for achieving greater financial autonomy and access to resources^47^.

The higher education sector in Pakistan also reflects these gender-based disparities. Studies on Pakistani university faculty have found that gender disparities are most pronounced at lower faculty levels, indicating systemic gender imbalances across educational institutions. This contributes to the wider issue of gender inequality in Pakistan, where women show lower involvement in financial activities, further exacerbating the gender gap in financial inclusion^12,48^.

### Research gap in financial inclusion in Pakistan

Although there has been considerable research on financial inclusion in Pakistan, there remains a notable gap, especially in gender-specific analyses. The nexus between financial inclusion and economic growth exhibits a positive trend, but the specific impacts on women are not sufficiently studied. Studies have pointed towards the importance of implementing financial inclusion strategies that empower women, suggesting a need for more focused research in this area. In addition, analyses highlighting the gender disparity in financial inclusion underline the considerable inequalities encountered by women, calling for a detailed examination of these issues.

This gap in the literature underscores the urgency for more targeted research on gender disparities in financial inclusion in Pakistan. It is important to investigate the socio-economic and cultural factors contributing to women’s financial exclusion and to understand how policy interventions can effectively address these challenges.

### Conceptual framework for financial inclusion

The multidimensional nature of financial inclusion is shaped by principles of equitable access and socio-economic empowerment. Sen’s^29^ capability approach emphasizes the importance of capability and access for development, framing financial inclusion as a mechanism for augmenting individual capabilities and choices, which aligns with the objectives of the Sustainable Development Goals, particularly SDG 1 (No Poverty), SDG 5 (Gender Equality), and SDG 10 (Reduced Inequalities). This perspective is further supported by the work of Robeyns^49^, who argues that the capability approach provides a comprehensive framework for assessing gender inequalities and their impact on development outcomes. However, the application of these theoretical frameworks to the study of gender disparities in financial inclusion in the specific context of Pakistan remains limited. This study bridges this theoretical gap by examining how the key tenets of the capability approach and behavioral economics can help explain the observed gender differences in financial access and usage in Pakistan, and how these theories can inform the development of more effective and gender-sensitive financial inclusion policies and interventions.

Behavioral economics, as studied by Karlan and Zinman^30^, enriches this understanding by examining how psychological and cognitive factors influence financial behavior, informing strategies to enhance engagement with financial services. Recent studies by Strömbäck, et al.^50^ and Ellili^51^ have further expanded on the role of behavioral factors in shaping financial decision-making and inclusion, particularly among women and marginalized communities.

The technological sphere, particularly the emergence of digital financial services, is revolutionizing the landscape of financial inclusion, with a marked impact in developing nations^52^. Recent research by Ozili^53^ and Arner et al.^54^ has emphasized the transformative potential of digital finance in promoting financial inclusion and reducing gender disparities. In addition, the growing body of literature on fintech and its impact on financial inclusion has explored the role of regulatory sandboxes, innovation hubs, and other enabling regulatory frameworks in fostering fintech development and facilitating the entry of new players that can contribute to closing the financial inclusion gap^55^. The regulatory and policy environment also play significant role in shaping the accessibility of financial services, especially for underserved communities^56^. Studies by Arun and Kamath^57^ and Agarwal, et al.^58^ have underscored the importance of inclusive financial regulations and policies in fostering equitable access to financial services.

**Figure 1:**
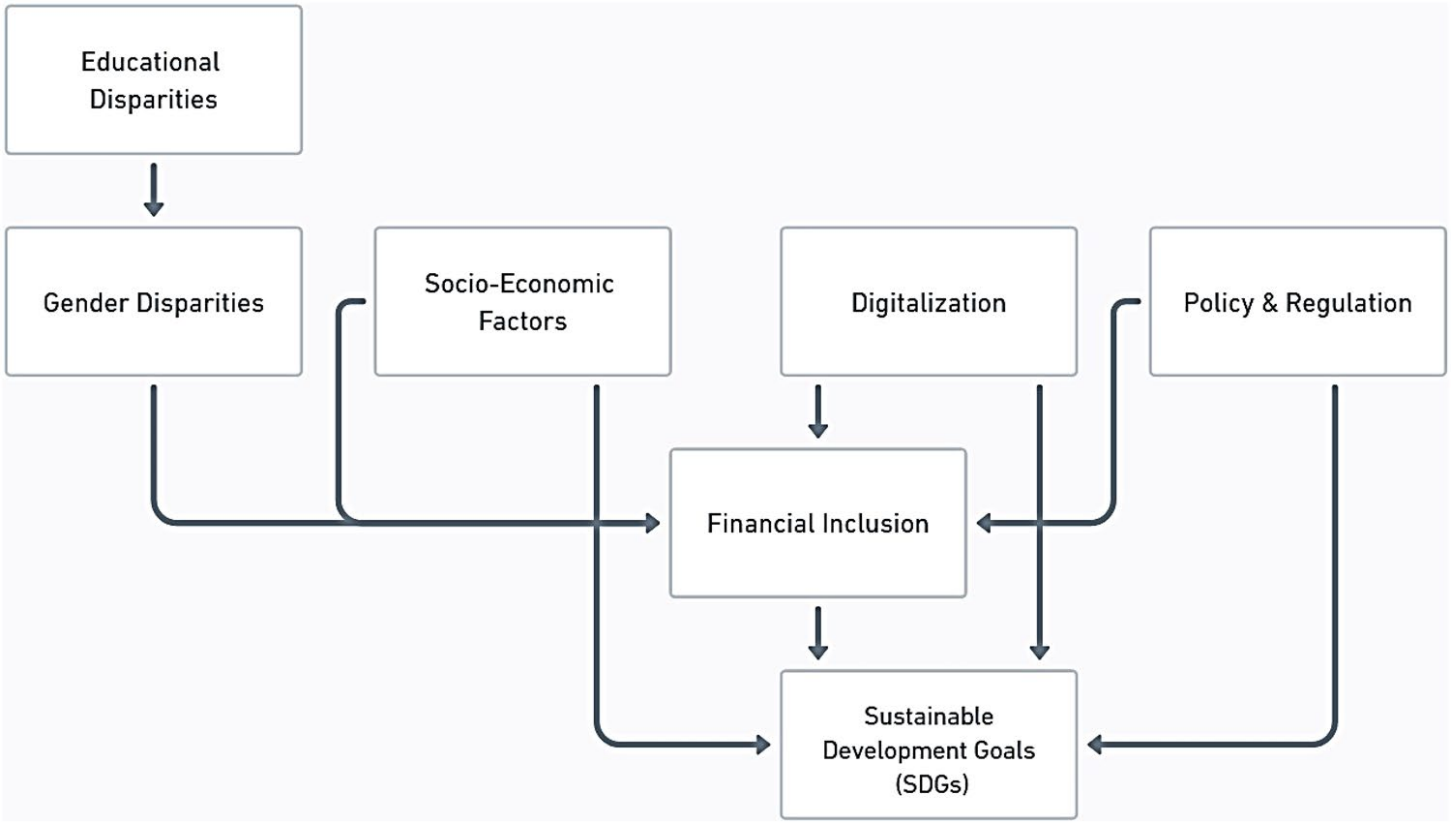
Conceptual framework of factors influencing financial inclusion and their impact on SDGs.

These diverse yet interconnected aspects form the foundation for developing comprehensive financial inclusion strategies in contexts such as Pakistan. Figure 1 illustrates how factors like gender and education disparities, economic conditions, digital finance, and policy shape financial inclusion and its effects on achieving SDGs in Pakistan.

## Materials and methods

The following section presents a detailed explanation of the materials and methods employed in this study, providing a clear rationale for the methodological decisions made and demonstrating the study’s adherence to best practices in financial inclusion research.

### Data sources

The primary data source for this study is the Global Findex 2021 dataset, a comprehensive and internationally recognized database that provides indicators measuring the use of financial services across 175 economies, including Pakistan. Developed by the World Bank in partnership with Gallup, Inc., the Global Findex is based on nationally representative surveys of more than 150,000 adults aged 15 and above, conducted between March 2020 and December 2021. The surveys use a multi-stage stratified random sampling methodology which is stratified by location (urban/rural) and region/province to ensure representativeness and account for the diversity of the population within each country^14^.

The Global Findex 2021 dataset is particularly well-suited for this research due to its extensive coverage of financial inclusion indicators, which encompass account ownership, savings and borrowing behaviors, digital payment methods, and financial resilience. The dataset’s granularity allows for a detailed examination of financial access and usage across various demographic groups, including gender, age, income, and education levels^14^.

Moreover, the Global Findex 2021 dataset provides the most up-to-date information on financial inclusion, capturing the impact of the COVID-19 pandemic on individuals’ financial behaviors and resilience. This timely data is essential for understanding the current state of financial inclusion in Pakistan and the potential effects of the pandemic on gender disparities in access to and use of financial services.

The survey data specific to Pakistan includes responses from 1,002 individuals, with an equal representation of men and women, ensuring the data’s suitability for gender-disaggregated analysis. The sampling methodology employed is stratified random sampling, with stratification criteria based on urban and rural regions, as well as the provinces of Punjab, Khyber Pakhtunkhwa, Sindh, and Balochistan. This approach ensures a representative sample of the Pakistani population, with sampling weights adjusted to accurately reflect the population distribution according to the 2017 census data^59^.

During the data cleaning process, responses recorded as ‘Do not know’ or ‘Refused to answer’ were treated as missing values to ensure the integrity of the data analysis. This procedure was applied consistently across all relevant variables, resulting in a slightly smaller sample size for some variables but maintaining the overall robustness of the dataset.

The robustness and reliability of the Global Findex 2021 dataset are further reinforced by the World Bank’s rigorous data collection and validation processes. The use of standardized questionnaires, trained interviewers, and quality control measures ensures the consistency and accuracy of the data across countries and over time. This methodological rigor allows for meaningful comparisons and analysis of financial inclusion trends, both within Pakistan and in relation to other economies.

### Variable definitions

The variables used in the regression analysis are detailed in Table 1, which provides a descriptive summary including mean and standard deviation for each variable. The dependent variables, ownership, and usage of financial products, are binary in nature, taking the value of 1 if the respondent owns or uses any financial product and 0 otherwise. This bi-variate nature of the dependent variables necessitates the use of logistic regression, which is specifically designed for analyzing dichotomous outcomes^60,61^.

**Table 1:** Variable definitions and summary statistics for the regression models.

Variables	Description	Type	Mean	Standard deviation
Dependent variables				
Ownership of financial products	Whether the respondent owns any financial product	Binary	0.24	0.43
Usage of financial products	Usage of any financial product	Binary	0.58	0.49
Independent variables				
Gender	1 for female and 0 for male respondents	Categorical	0.50	0.50
Young	1 if respondent’s age is 15–24 years, 0 otherwise	Binary	0.26	0.44
Middle	1 if respondent’s age is between 24 and 59, 0 otherwise	Binary	0.60	0.49
Old	If respondents is 50 years or older	Binary	0.15	0.35
Edu_primary	1 if the respondent has completed primary education, otherwise 0	Binary	0.59	0.49
Edu_secondary	1 if the respondent has completed secondary education, otherwise 0		0.33	0.47
Edu_tertiary	1 if the respondent has completed tertiary education, otherwise 0		0.08	0.27
Inc_q1	Represents the poorest 20% (1st quintile), otherwise 0	Ordinal/categorical	0.16	0.36
Inc_q2	Represents the second 20% (2nd quintile), otherwise 0		0.15	0.36
Inc_q3	Represents to the middle 20% (3rd quintile), otherwise 0		0.20	0.40
Inc_q4	Represents the fourth 20% (4th quintile), otherwise 0 (fourth 20%), 0 otherwise		0.21	0.41
Inc_q5	Refers to the richest 20% (5th quintile), otherwise 0		0.28	0.45
Employment	1 if the respondent is part of the workforce, otherwise 0	Binary	0.44	0.50
Location	1 for urban residents, 0 for rural dwellers	Categorical	0.63	0.48
*N*			1002	

The ownership of financial products variable is constructed based on three key indicators: having a financial account, having an account at a financial institution, and having a mobile money account. These indicators were combined to create a comprehensive measure of financial product ownership.

The usage of financial products variable is composed of several sub-variables capturing different aspects of financial behavior, such as depositing money, withdrawing money, making bill payments, online shopping, borrowing, and saving. These sub-variables were derived from a combination of relevant indicators in the Global Findex dataset and then aggregated to create the overall usage variable.

The independent variables capture a range of demographic and socioeconomic factors that are expected to influence financial inclusion. The selection of these variables is informed by the existing literature on the determinants of financial inclusion and their relevance to the specific research questions of the study.

Key independent variables include gender, age, educational attainment, income level, employment status, and urban/rural location. These variables are expected to have varying associations with financial inclusion based on the literature and the specific context of Pakistan.

### Econometric approach and model specification

The econometric approach employed in this study is logistic regression analysis, which is well-suited for examining the determinants of financial inclusion given the binary nature of the dependent variables. Logistic regression is widely used in financial inclusion research for its ability to model dichotomous outcomes and provide estimates of the probability of an event occurring based on a set of independent variables^62,63^.

The model specification is as follows:

$$log\frac{P({y}_{i} =1}{1-P\left({y}_{i} =1\right)}= {\beta }_{0}+{\beta }_{1}{Gender}_{i}+{\beta }_{2}{Age}_{i}+{\beta }_{3}{Education}_{i}+{\beta }_{4}{Income}_{i}+{\beta }_{5}{Employment}_{i}+{\beta }_{6}{Location}_{i}+{\varepsilon }_{i}$$

where *P*(*y*_*i*​_ = 1) represents the probability of owning or using financial products, and *β*_1_​ to *β*_6_​ are the coefficients to be estimated. The coefficients represent the change in the log odds of the dependent variable for a one-unit change in the corresponding independent variable, holding other variables constant. ${\varepsilon }_{i}$ is the error term, which captures the unexplained variation in the dependent variable.

The process of model selection involved the consideration of alternative model specifications and the use of established criteria, such as the Akaike Information Criterion (AIC) and the Bayesian Information Criterion (BIC), to arrive at the final model^64^. The chosen model specification was found to provide the best balance between model fit and parsimony, while also aligning with the theoretical foundations and research questions of the study.

To ensure the robustness of the results, several diagnostic tests are conducted. First, the variance inflation factor (VIF) is calculated for each independent variable to check for multicollinearity. VIF values greater than 5 would indicate a high degree of multicollinearity, which could bias the coefficient estimates. Second, the Hosmer–Lemeshow test is performed to assess the goodness of fit of the logistic regression model. A non-significant result (p > 0.05) suggested that the model fits the data well.

In addition to these diagnostic tests, we also conducted sensitivity analyses to assess the stability of the results under different assumptions or model specifications. This involved re-estimating the model with alternative variable definitions, subsamples of the data, and different estimation techniques (e.g., probit regression) to ensure that the main findings were robust to these variations^65^.

Furthermore, we took steps to ensure the reliability and validity of the data used in the analysis. This included checking for consistency across variables, handling missing values appropriately (as described in the data sources section), and examining the data for potential outliers or influential observations that could unduly influence the results. Any issues identified were carefully addressed to maintain the integrity of the data and the validity of the findings.

## Results

### Demographic characteristics

Table 2 presents the demographic characteristics of the respondents from the Global Findex 2021 dataset, encompassing a total of 1002 individuals. The table provides a summary of key demographic variables including gender distribution, age, educational attainment, income levels, employment status, and urban or rural residency. These indicators are fundamental to the subsequent analysis of financial inclusion patterns among the surveyed population.

**Table 2:** Summary statistics of demographic characteristics of respondents in the Global Findex 2021 dataset.

Demographic characteristics	Mean	Std. deviation
Gender (0 = male, 1 = female)	0.50	0.50
Respondent age	33.95	12.33
Primary education attainment	0.59	0.49
Secondary education attainment	0.33	0.47
Tertiary education attainment	0.08	0.27
Respondent education level	1.50	0.64
Economic Bracket—Lowest 20%	0.16	0.36
Economic Bracket—Second 20%	0.15	0.36
Economic Bracket—Middle 20%	0.20	0.40
Economic Bracket—Fourth 20%	0.21	0.41
Economic Bracket—Top 20%	0.28	0.45
Respondent is in workforce	0.44	0.50
Location (0 = urban, 1 = rural)	0.37	0.48
No. of observations	1002	

The data reveals a balanced gender distribution among respondents (mean = 0.50), suggesting an equal representation of males and females. The average age of the respondents is approximately 34 years, indicating a predominantly young to middle-aged population. Education levels are varied, with a significant proportion having completed only primary education (mean = 0.59). This educational background plays a significant role as it influences financial literacy and, consequently, financial inclusion.

Income distribution, categorized into quintiles, indicates economic diversity among the respondents. The workforce participation rate is less than half (mean = 0.44), highlighting a potentially significant segment of the population outside the formal employment sector. Additionally, the urban–rural divide, with a majority residing in urban areas (mean = 0.37), points to differences in access to financial services.

### Financial inclusion in Pakistan: account ownership, digital financial services, and savings behavior

Table 3 presents a comprehensive analysis of financial inclusion metrics in Pakistan, encompassing account ownership, digital financial services usage, and savings behavior. The study reveals that 24% of the respondents have an account, with a smaller proportion, 19%, holding an account at a formal financial institution, and 9% using mobile money accounts. This data suggests a moderate level of financial services penetration. Digital financial service usage is relatively low; 21% of respondents access their accounts using mobile or internet banking, yet activities such as checking balances or making in-store purchases via mobile are less common. In contrast, credit and debit card usage among respondents is minimal, with only 3% owning a credit card and 24% using a debit card. The utilization of these cards for in-store purchases remains limited, indicating a nascent stage in the adoption of card-based transactions.

**Table 3:** Financial inclusion in Pakistan (2021 Global Findex Database).

	No. of Obs	Mean	Std. Dev
Account ownership			
Possession of any financial account	1002	0.24	0.43
Account ownership at a financial institution	1002	0.19	0.39
Mobile money account ownership	1002	0.09	0.29
Digital financial services usage			
Accessing account via mobile phone or internet	145	0.21	0.41
Checking account balance via digital platforms	142	0.20	0.40
In-store purchases via mobile phone	1002	0.01	0.10
Online bill payments	999	0.07	0.25
Online money transfers to friends or relatives	998	0.07	0.26
Online purchases	996	0.01	0.10
Credit and debit card usage			
Debit card usage	110	0.24	0.43
In-store debit card usage	26	0.27	0.45
Credit card ownership	144	0.03	0.18
Credit card usage	5	0.80	0.45
In-store credit card usage	4	1.00	0.00
Deposits and withdrawals			
Made any deposit into the account	141	0.62	0.49
Withdrew from the account	144	0.76	0.43
Withdrew from the account two or more times per month	107	0.61	0.49
Used account to store money	145	0.60	0.49
Saving behavior			
Saved for old age	995	0.07	0.25
Saved through a financial institution account	991	0.03	0.17
Saved through a mobile money account	126	0.08	0.27
Participated in an informal savings club	993	0.07	0.26

The analysis of deposits, withdrawals, and savings behaviors provides additional insights. A significant portion of the respondents, 62%, actively make deposits, and 76% perform withdrawals, with over half withdrawing funds multiple times a month. These patterns point to active use of accounts for transactional purposes. However, savings behavior appears modest; only a small percentage of respondents save for old age or use formal and informal saving instruments. This divergence between transactional use and savings illustrates the challenges and opportunities in enhancing comprehensive financial inclusion. The data underscores the need for targeted interventions to promote not only account ownership but also the broader spectrum of financial services, including digital and card-based platforms, to foster more inclusive financial participation in Pakistan.

### Financial inclusion and gender disparities

The examination of gender disparities in financial inclusion within Pakistan, detailed in Table 4, uncovers distinct differences in account ownership and digital financial service usage between male and female respondents. This analysis, grounded in the Global Findex 2021 dataset, is vital in understanding how financial inclusion varies across genders in the context of a developing country like Pakistan.

**Table 4:** Gender differences in account ownership and digital financial services usage.

Variable	Male		Female	
	No. of Obs	Mean	No. of Obs	Mean
Account ownership				
Possession of any financial account	499	0.34***	503	0.13
Account ownership at a financial institution	499	0.27***	503	0.11
Mobile money account ownership	499	0.15***	503	0.03
Digital financial services usage				
Accessing account via mobile phone or internet	118	0.25**	27	0.07
Checking account balance via digital platforms	115	0.22	27	0.15
In-store purchases via mobile phone	499	0.01***	503	0.01
Online bill payments	499	0.09**	500	0.04
Online money transfers to friends or relatives	498	0.12***	500	0.03
Online purchases	499	0.01	497	0.01
Credit and debit card usage				
Debit card usage	97	0.22	13	0.38
In-store debit card usage	21	0.33**	5	0.00
Credit card ownership	117	0.04**	27	0.00
Credit card usage	5	0.80		
In-store credit card usage	4	1.00		
Deposits and withdrawals				
Made any deposit into the account	114	0.69***	27	0.33
Withdrew from the account	117	0.80**	27	0.56
Withdrew from the account two or more times per month	92	0.63	15	0.47
Used account to store money	118	0.64	27	0.44
Saving behavior				
Saved for old age	496	0.06	499	0.07
Saved through a financial institution account	495	0.05***	496	0.01
Saved through a mobile money account	103	0.09	23	0.04
Participated in an informal savings club	495	0.07	498	0.07

*p < 0.05; **p < 0.01; ***p < 0.001 (t-test).

Our analysis reveals a significant gender gap in account ownership. Males significantly outpace females in possessing any form of a financial account: 34% of males hold an account compared to only 13% of females. This disparity extends to specific types of accounts; for instance, 27% of males have accounts at financial institutions versus 11% for females, and 15% of males own mobile money accounts against a mere 3% for females. These figures highlight the gender disparity in basic financial access, indicating a critical area for policymakers focused on improving financial inclusion for women.

The utilization of digital financial services further emphasizes the gender divide. Male respondents demonstrate a higher propensity to engage with digital platforms for financial transactions, including accessing accounts (25% for males vs. 7% for females) and checking account balances (22% for males vs. 15% for females). However, activities such as making in-store purchases via mobile phones or conducting online bill payments do not exhibit significant gender differences, indicating areas where women’s participation in digital finance is comparable to men’s.

Credit and debit card usage also reflects gender-based variations. While 22% of male respondents reported using a debit card, a higher percentage (38%) of females indicated the same. Credit card ownership, however, remains relatively low across both genders, with a slight skew towards male respondents. In terms of transactional behavior, males again lead in depositing into (69% for males vs. 33% for females) and withdrawing from accounts (80% for males vs. 56% for females), implying more active engagement with financial services.

The savings behavior of respondents does not demonstrate a marked gender difference. Similar proportions of males and females save for old age, use various saving instruments, including accounts at financial institutions, mobile money accounts, and informal savings clubs. This aspect of financial behavior suggests that, despite discrepancies in account ownership and digital service usage, the propensity to save is relatively uniform across genders.

**Figure 2:**
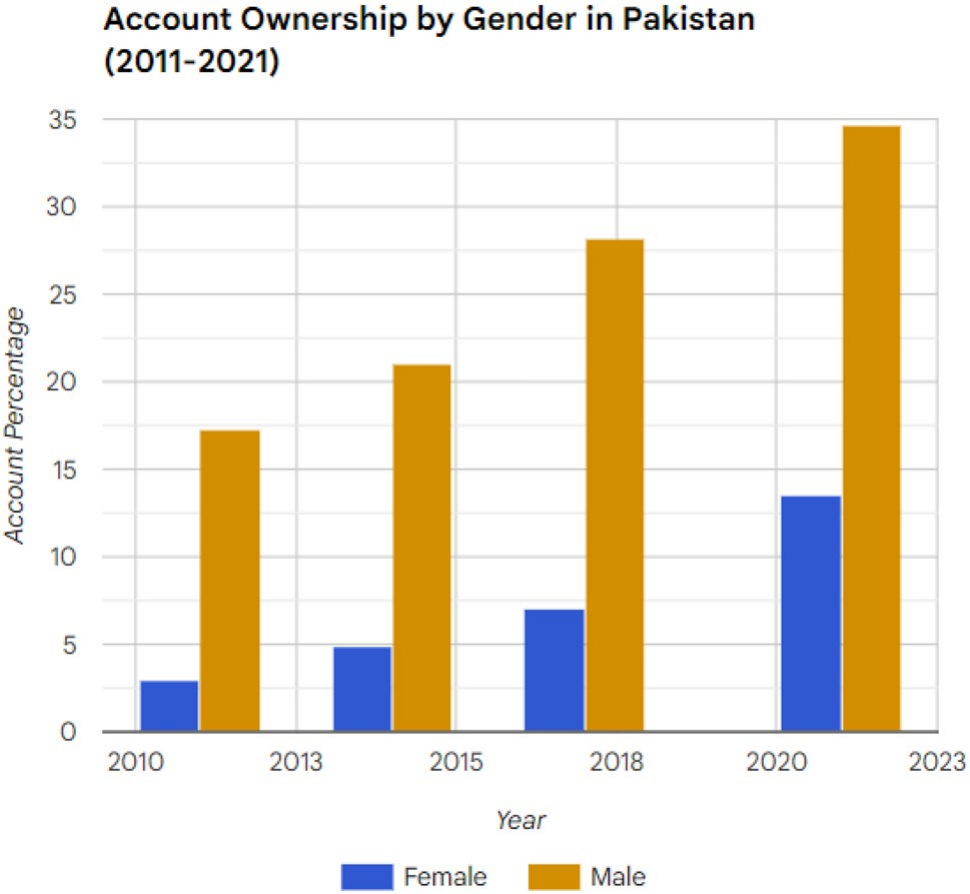
Trends in account ownership in Pakistan (2011–2021).

The data since 2011 shows the gender disparities in account ownership are persistent. In Fig. 2, we observe the progression of account ownership among the adult population in Pakistan across a decade, differentiated by gender. From 2011 to 2021, there is a noticeable upward trajectory in the percentage of both males and females who own an account, suggesting an overall positive trend in financial inclusion within the country.

A closer analysis reveals a persistent gender gap, with male account ownership consistently surpassing that of females throughout the observed period. The data indicates a nearly sixfold increase in female account ownership from 2011 to 2021, an encouraging sign of improved financial empowerment among women. However, the male account ownership in the same timeframe shows a two-fold increase, reinforcing the presence of a gender disparity.

### Gender disparities in determinants of financial products ownership

Table 5 presents the logistic regression analysis on the determinants of financial product ownership in Pakistan, examining the influence of various demographic and socioeconomic factors. The logistic regression results for the entire sample indicate that youth and educational attainment up to the secondary level are associated with a reduced likelihood of owning financial products. Young individuals are 10% less likely to own financial products, indicating that age has a significant impact on financial inclusion. Primary and secondary education attainment reduces the probability of owning financial products by 32% and 15%, respectively, highlighting the importance of advanced education in facilitating access to financial services. Conversely, being employed and living in urban areas significantly increases the probability of owning financial products, by 20% and 9% respectively. These findings underscore the role of economic activity and urban infrastructure in enhancing financial access.

**Table 5:** Determinants of ownership of financial products.

	Full Sample		Females		Males	
	Beta	Marginal effects	Beta	Marginal effects	Beta	Marginal effects
fin_inc						
gender (d)	− 0.725***	− 0.114***	[…]	[…]	[…]	[…]
	(0.208)	(0.0324)				
young (d)	-0.736***	− 0.103***	0.116	0.00665	− 1.355***	− 0.105***
	(0.282)	(0.0351)	(0.426)	(0.0251)	(0.345)	(0.0217)
middle (d)	− 0.247	− 0.0393	− 0.108	− 0.00611	− 0.399	− 0.0402
	(0.235)	(0.0379)	(0.393)	(0.0224)	(0.266)	(0.0277)
edu_prim (d)	− 1.883***	− 0.322***	− 0.593	− 0.0351	− 2.119***	− 0.250***
	(0.285)	(0.0513)	(0.467)	(0.0292)	(0.314)	(0.0445)
edu_sec (d)	− 1.100***	− 0.155***	− 0.331	− 0.0177	− 1.072***	− 0.0927***
	(0.277)	(0.0357)	(0.463)	(0.0236)	(0.297)	(0.0243)
inc_q1 (d)	0.0372	0.00589	0.379	0.0239	− 0.0758	− 0.00725
	(0.263)	(0.0419)	(0.407)	(0.0286)	(0.313)	(0.0294)
inc_q2 (d)	− 0.424	− 0.0607*	− 0.0381	− 0.00211	− 0.597*	− 0.0497**
	(0.284)	(0.0368)	(0.453)	(0.0248)	(0.338)	(0.0238)
inc_q3 (d)	− 0.291	− 0.0433	0.0173	0.000975	− 0.393	− 0.0351
	(0.248)	(0.0349)	(0.410)	(0.0232)	(0.287)	(0.0234)
inc_q4 (d)	− 0.0403	− 0.00628	0.294	0.0178	− 0.225	− 0.0209
	(0.230)	(0.0356)	(0.366)	(0.0238)	(0.265)	(0.0234)
emp (d)	0.718***	0.116***	− 0.593**	− 0.0325**	1.832***	0.204***
	(0.201)	(0.0331)	(0.282)	(0.0148)	(0.212)	(0.0239)
location (d)	0.612***	0.0917***	0.495*	0.0264*	0.595***	0.0549***
	(0.180)	(0.0254)	(0.294)	(0.0147)	(0.211)	(0.0184)
Const	0.176 (0.381)	[…]	2.437*** (0.608)	[…]	− 0.859** (0.409)	[…]
N	1002					
LR chi2	164.64	[…]	12.32	[…]	201.36	[…]
Prob > chi2	0.0000	[…]	0.2644	[…]	0.0000	[…]
Pseudo R2	0.1502	[…]	0.0256	[…]	0.2191	[…]

Standard errors in parentheses; (d) stands for discrete change of dummy variable from 0 to 1; *p < 0.1, **p < 0.05, ***p < 0.01.

When segregated by gender, the analysis reveals distinct patterns. For females, neither primary nor secondary education significantly impacts financial product ownership, diverging from the overall sample trend. This disparity might reflect the broader socio-cultural context in Pakistan, where women’s education does not linearly translate into financial empowerment. Interestingly, employment status shows contrasting effects between genders. For males, employment enhances the likelihood of owning financial products (11.6% and 20.4%), while it decreases it for females by 3.25%. This finding could be attributed to the types of employment opportunities available to women, which may not adequately translate into financial inclusion. Urban residency positively influences ownership for both genders, albeit with a greater impact on females.

These results show that age and education level pose significant challenges to financial inclusion, particularly for females. Employment and urban residency are key facilitators of financial inclusion, but their impact varies notably between genders. The findings reveal that employment paradoxically seems to deter female ownership of financial products, a phenomenon warranting further investigation into the quality and nature of employment for women in Pakistan.

### Gender disparities in determinants of financial products usage

Table 6 provides a detailed logistic regression analysis on the determinants of financial product usage in Pakistan, with an emphasis on how demographic and socioeconomic attributes influence this usage differently across genders. The results, both for the total sample and when disaggregated by gender, provide a comprehensive view into the factors shaping financial behavior in the country.

**Table 6:** Determinants of usage of financial products.

Variables	Full Sample		Females		Males	
	Beta	Marginal effects	Beta	Marginal effects	Beta	Marginal effects
usage_fin						
gender (d)	0.280*	0.0677*				
	(0.169)	(0.0407)				
young (d)	− 0.259	− 0.0632	0.810***	0.165***	− 1.200***	− 0.171***
	(0.222)	(0.0546)	(0.267)	(0.0578)	(0.269)	(0.0317)
middle (d)	− 0.152	− 0.0366	0.728***	0.130***	− 0.881***	− 0.156***
	(0.196)	(0.0472)	(0.246)	(0.0421)	(0.228)	(0.0416)
edu_prim (d)	− 1.892***	− 0.417***	− 0.211	− 0.0396	− 1.788***	− 0.324***
	(0.348)	(0.0645)	(0.315)	(0.0597)	(0.291)	(0.0541)
edu_sec (d)	− 1.328***	− 0.319***	− 0.361	− 0.0652	− 0.804***	− 0.126***
	(0.350)	(0.0793)	(0.319)	(0.0556)	(0.284)	(0.0413)
inc_q1 (d)	− 0.340	− 0.0836	− 0.478*	− 0.0815**	0.0871	0.0150
	(0.215)	(0.0534)	(0.256)	(0.0398)	(0.258)	(0.0452)
inc_q2 (d)	− 0.538**	− 0.133**	− 0.0856	− 0.0157	− 0.862***	− 0.122***
	(0.217)	(0.0538)	(0.251)	(0.0454)	(0.296)	(0.0338)
inc_q3 (d)	− 0.0308	− 0.00746	− 0.0139	− 0.00258	− 0.00269	− 0.000455
	(0.202)	(0.0490)	(0.231)	(0.0428)	(0.237)	(0.0401)
inc_q4 (d)	− 0.0707	− 0.0172	0.127	0.0240	− 0.197	− 0.0323
	(0.198)	(0.0482)	(0.223)	(0.0430)	(0.231)	(0.0365)
emp (d)	− 0.332**	− 0.0804**	− 2.416***	− 0.409***	1.745***	0.310***
	(0.168)	(0.0407)	(0.198)	(0.0253)	(0.170)	(0.0287)
location (d)	0.168	0.0408	− 0.208	− 0.0393	0.528***	0.0859***
	(0.138)	(0.0336)	(0.161)	(0.0308)	(0.175)	(0.0271)
Const	2.123*** (0.410)	[…]	− 0.257 (0.389)	[…]	− 0.0789 (0.359)	[…]
N	1002	[…]	1002	[…]	1002	[…]
LR Chi2	79.97	[…]	230.75	[…]	222.45	[…]
Prob > chi2	0.0000	[…]	0.0000	[…]	0.0000	[…]
Pseudo R2	0.0586	[…]	0.1861	[…]	0.1905	[…]

Standard errors in parentheses; (d) stands for discrete change of dummy variable from 0 to 1; *p < 0.1, **p < 0.05, ***p < 0.01.

In the aggregated sample, gender plays a significant role, with males more likely to use financial products. This gender disparity could be rooted in the socio-cultural landscape of Pakistan, where financial activities and opportunities often differ markedly between men and women. Age also plays a critical role; younger individuals have a reduced likelihood of using financial products, possibly indicating gaps in financial literacy or accessibility among this demographic. Interestingly, both primary and secondary education levels are negatively correlated with product usage, suggesting that higher educational attainment might not directly translate to increased financial engagement, perhaps due to the nature of the education system or societal norms that limit the application of formal education in financial matters.

Income levels present a nuanced narrative. Lower income quintiles—representing the poorer segments of society—demonstrate a reduced probability of using financial products, while higher income brackets do not exhibit a significant impact. This pattern could reflect the barriers faced by lower-income individuals in accessing financial products or their lack of trust or familiarity with these services. The negative effect of employment on product usage is counterintuitive and suggests that the type of employment or income stability might be more influential than mere employment status. Urban residency surprisingly shows no significant effect, indicating that urban–rural differences might not be as significant in determining financial product usage, contrary to common assumptions.

When examining the gender-segregated data, distinct patterns emerge. For women, employment significantly reduces the likelihood of using financial products, highlighting the unique challenges they face in accessing financial services. This could be due to the nature of employment for women in Pakistan, often characterized by informal or unstable jobs that do not facilitate financial inclusion. Conversely, for men, employment appears to boost the likelihood of using financial products, suggesting that economic engagement plays a different role in men’s financial behavior.

## Discussion

The findings of this study reveal significant gender disparities in financial inclusion in Pakistan, aligning with Sen’s^29^ capability approach, which emphasizes the importance of access and choice in enhancing individual capabilities and fostering development. Women’s limited access to and usage of financial services restricts their ability to make choices about their economic well-being, hindering their overall capabilities and perpetuating gender inequalities. This resonates with the work of^66^, who highlight the role of resources and agency in achieving women’s empowerment. The observed gender gap is consistent with findings from other developing countries, suggesting that these disparities are not unique to Pakistan but reflect broader global patterns of gender inequality in access to financial resources^14,67^. Furthermore, our analysis of the determinants of financial inclusion, focusing on factors such as age, education, income, employment, and location, provides a comprehensive understanding of the barriers and enablers to financial access and usage in Pakistan.

The pronounced gender gap in financial inclusion observed in our study aligns with the findings of Chundakkadan and Sasidharan^68^ Swamy^69^, who highlight the persistent gender inequalities in access to finance in South Asian countries. However, our study extends beyond the mere identification of gender disparities by examining the specific ways in which socio-economic factors interact with gender to shape financial inclusion outcomes in Pakistan. For instance, while Fungáčová and Weill^63^ and Zins and Weill^70^ have identified the role of income and education in determining financial inclusion in China and Africa, respectively, our study reveals the distinct ways in which these factors influence financial access and usage for men and women in Pakistan.

A key finding of our study is the differential impact of employment on financial inclusion for men and women in Pakistan. While employment is generally associated with higher levels of financial access and usage, we find that this effect is less pronounced for women. This observation is consistent with the findings of Aterido, et al.^71^, who note that the gender gap in financial inclusion persists even among the employed in several developing countries. We argue that this disparity in the employment-financial inclusion nexus for men and women in Pakistan is likely rooted in the country’s broader gender inequalities in the labor market, where women often face limited access to formal and well-paying jobs^25,72^.

Our study also draws attention to the role of societal norms and cultural practices in shaping financial inclusion outcomes, particularly for women in Pakistan. The complex relationship between women’s education and their financial autonomy, as observed in our findings, resonates with the work of Demirguc-Kunt, Klapper and Singer^24^ and Fanta and Mutsonziwa^73^, who emphasize the influence of social and cultural factors on women’s financial inclusion in developing countries. While education can expand women’s capabilities and agency^29^, deeply ingrained patriarchal norms and gender-based discrimination may still hinder their ability to fully leverage educational gains for greater financial autonomy and inclusion^74,75^.

The adoption of digital financial services, an emerging avenue for enhancing financial inclusion, also exhibits gender disparities in our study, mirroring the findings of Ibtasam et al.^16^ in the context of Pakistan. We posit that the lower uptake of digital financial services among women in Pakistan may be attributed to a combination of factors, including limited access to technology, lower levels of digital literacy, and societal norms that restrict women’s engagement with digital platforms^16,76^. However, the growth of Fintech in Pakistan has the potential to address these barriers, as Aleemi, et al.^77^ demonstrate with their evidence of Fintech’s positive impact on financial inclusion.

Comparing our findings with international contexts, we observe parallels in the gender disparities in financial inclusion across regions such as Sub-Saharan Africa^24,71^ and South Asia^67,69^. However, our study’s strength lies in its detailed analysis of factors specifically affecting gender disparities in financial inclusion within Pakistan. We use the latest Global Findex 2021 data and robust quantitative methods to provide a detailed analysis of the factors influencing the gender gap in financial access and usage. This analysis offers a better understanding of the challenges and potential solutions for promoting gender equality in Pakistan’s financial system.

Finally, our study provides a comprehensive, data-driven analysis of the factors driving gender disparities in financial inclusion within Pakistan. It considers the complex relationships between socio-economic, cultural, and institutional barriers to financial access and use. By placing our findings in a global context, we enhance the understanding of challenges and potential solutions for gender equality in financial inclusion within Pakistan and other developing nations. These findings can guide policymakers and financial institutions seeking to achieve the SDGs by designing interventions that address the multifaceted nature of gender disparities, leading to more inclusive and equitable growth.

## Conclusions, policy implications, and research limitations

### Conclusions

This study provides a comprehensive examination of the determinants of gender disparities in financial inclusion in Pakistan, utilizing the latest Global Findex 2021 data and employing rigorous quantitative methods. The findings reveal significant gender gaps in both access to and usage of financial services, with women being substantially less likely than men to own formal financial accounts or engage with digital financial services. The analysis of the socio-economic factors influencing these disparities underscores the complex interaction of individual characteristics, such as age, education, income, and employment, with broader structural and societal barriers that disproportionately affect women’s financial inclusion.

The results of this study contribute to the growing body of literature on gender and financial inclusion, specifically enriching the understanding of Sen’s capability approach. In addition, the findings provide important information on the specific challenges and opportunities for promoting women’s financial empowerment in Pakistan. By situating the findings within the broader global discourse on financial inclusion and the Sustainable Development Goals (SDGs), this research emphasizes the critical role of addressing gender disparities in financial access and usage for achieving inclusive growth and development.

### Policy implications

The findings of this study have significant implications for policymakers and financial institutions seeking to promote gender equality in financial inclusion in Pakistan. The results indicate the need for targeted interventions and policies that address the multi-dimensional nature of the barriers faced by women in accessing and using financial services. This may involve initiatives aimed at improving women’s education and employment opportunities, as well as efforts to challenge discriminatory societal norms and practices that limit women’s financial autonomy.

Policymakers should focus on developing gender-sensitive financial inclusion strategies that take into account the specific needs and preferences of women, aligning with the recommendations of international organizations like the World Bank^52^ and the Alliance for Financial Inclusion (AFI). This may involve designing financial products and services that cater to women’s life cycles and economic activities, such as micro-loans for women entrepreneurs, savings products with flexible withdrawal options, and insurance schemes tailored to women’s health risks. Successful examples from other developing countries, such as the mobile money revolution in Kenya that empowered women through financial access^78^, can provide valuable insights for designing context-specific interventions in Pakistan. In addition, incorporating insights from behavioral economics, such as nudges and simplified processes, can encourage greater uptake of financial services among women, as demonstrated by various field experiments in developing countries^79^.

Financial institutions can play a key role in promoting gender equality in financial inclusion by adopting gender-responsive policies and practices, such as setting targets for women’s representation in leadership positions and implementing gender-sensitive customer service and grievance redressal mechanisms. Collaborations between policymakers, financial institutions, and civil society organizations can help foster an enabling environment for women’s financial empowerment, through initiatives such as awareness campaigns, capacity building programs, and policy advocacy.

### Limitations

While this study provides important information on the determinants of gender disparities in financial inclusion in Pakistan, it is essential to acknowledge its limitations. Firstly, the analysis relies on cross-sectional data from the Global Findex 2021 survey, which limits the ability to establish causal relationships between the variables examined. Secondly, due to data availability and scope, the study focuses primarily on access and usage of financial services. Incorporating additional dimensions, such as availability, quality of services, and barriers would provide a more comprehensive assessment in future studies. Furthermore, the 2021 data may not fully reflect the impact of the COVID-19 pandemic on financial inclusion. Nevertheless, this study utilizes a robust and widely accepted dataset and employs appropriate statistical methods.

### Future research directions

The findings of this study open up several avenues for future research on gender and financial inclusion in Pakistan and other developing contexts. One potential area of investigation is the role of technology and digital financial services in bridging the gender gap in financial access and usage. Future studies could examine the specific barriers and enablers to women’s adoption of digital financial services, as well as the impact of digital financial inclusion on women’s economic empowerment and well-being.

## Data Availability

The data that support the findings of this study are freely available from The Global Findex Database website https://www.worldbank.org/en/publication/globalfindex.
